# The Sequential Change in Left Ventricular Function among Various Cardiovascular Diseases: A 12-Year Study

**DOI:** 10.3390/jpm12030415

**Published:** 2022-03-07

**Authors:** Sheng-Nan Chang, Jou-Wei Lin, Yi-Chih Wang, Cho-Kai Wu, Jun-Jack Cheng, Juey-Jen Hwang, Jiunn-Lee Lin, Fu-Tien Chiang, Yih-Sharng Chen, Ron-Bin Hsu, William Chen, Jin-Jer Chen, Wen-Pin Lien

**Affiliations:** 1 Division of Cardiology, Department of Internal Medicine, National Taiwan University College of Medicine and Hospital, Yun-Lin Branch, Yun-Lin City 640, Taiwan; p95421008@ntu.edu.tw (S.-N.C.); jouweilin@yahoo.com (J.-W.L.); 2 Division of Cardiology, Department of Internal Medicine, National Taiwan University College of Medicine and Hospital, Taipei City 100, Taiwan; med011@seed.net.tw (Y.-C.W.); chokaiwu@yahoo.com.tw (C.-K.W.); jueyhwang@ntu.edu.tw (J.-J.H.); jiunnlee@ntu.edu.tw (J.-L.L.); futienc@ntuh.gov.tw (F.-T.C.); jc8510@yahoo.com (J.-J.C.); 3 Division of Cardiology, Shin-Kong Wu-Ho Su Memorial Hospital, Taipei City 111, Taiwan; jackcheng126@gmail.com; 4 Division of Cardiology, Department of Internal Medicine, Fu-Jen Catholic University Hospital, Fu-Jen Catholic University, New Taipei City 243, Taiwan; 5 Division of Cardiovascular Surgery, Department of Surgery, National Taiwan University College of Medicine and Hospital, Taipei City 100, Taiwan; yschen1234@gmail.com (Y.-S.C.); ronbin@ntuh.gov.tw (R.-B.H.); 6 Nuliv Wellness Clinic, Taipei City 110, Taiwan; nuliv.marketing@gmail.com

**Keywords:** integrated score index, diastolic function, 2-dimensional echocardiography, tissue Doppler imaging, hypertension, elderly, coronary artery disease

## Abstract

Background: This 12-year study aimed to compare the longitudinal change in left ventricular diastolic dysfunction (LVDD) between healthy elderly, coronary artery disease (CAD), and hypertension (HTN) patients. Methods: From 2008 to 2020, 1476 patients were included, and 3181 echocardiography examinations were conducted. Finally, 130 participants (36 healthy elderly (79.39 ± 9.51 years old), 51 with CAD (68.31 ± 12.09 years old), and 43 with HTN (68.31 ± 12.09 years old)) with more than a 10-year follow-up period were included in the final analysis. Results: The change in diastolic function was different among these subjects according to the integrated score index (elderly vs. HTN, *p* = 0.01; CAD vs. HTN, *p* = 0.01), septal E/e′ ratio (elderly vs. HTN, *p* < 0.001; CAD vs. HTN, *p* = 0.01), lateral E/e′ ratio (elderly vs. HTN, *p* < 0.001; CAD vs. HTN, *p* < 0.001), and NYHA functional class (elderly vs. HTN, *p* = 0.03; CAD vs. HTN, *p* < 0.001). Additionally, per one-year increase in age, the integrated score index increased 0.2 in the healthy elderly, 0.15 in the CAD, and 0.06 in the HTN patients (all *p* < 0.05). Conclusion: Under aggressive treatment, diastolic function was relatively preserved in HTN subjects with aging in comparison with elderly and CAD subjects.

## 1. Introduction

Intrinsic cardiac aging is defined as age-related degeneration and decline in cardiac function, either left ventricular systolic or diastolic dysfunction (LVDD) [1]. For patients with heart failure, more than half have heart failure with a preserved ejection fraction (HFpEF), characterized by LVDD [2]. Even with advanced treatment, the rehospitalization and mortality rates are still very high for patients with HFpEF [2].

In addition to cardiac aging, other diseases such as coronary artery disease (CAD), hypertension (HTN), and physical deconditioning, appear to be critical in the onset of LVDD and its progression towards HFpEF [3]. Kuznetsova et al. showed that present baseline diastolic blood pressure (DBP) and the change in systolic blood pressure (SBP) over time predicted worsening of LVDD in the general population (*p* ≤ 0.014) [4]. However, data from a longitudinal comparison of diastolic function between CAD and HTN patients with aging are still sparse. This was the motivation for the longitudinal follow-up of left ventricular diastolic function among the healthy elderly and those with systemic diseases (CAD or HTN). We aimed for this study to be the longest follow-up of diastolic function, with the largest numbers of patients and oldest population, including the elderly, CAD, and HTN patients. We hypothesized that aggressive treatment in HTN patients might slow the deterioration of diastolic function with the aging process as compared with CAD patients.

## 2. Methods

### 2.1. Study Setting and Participants

In this single-center study, 1476 participants were enrolled initially for transthoracic echocardiography from January 2008 to September 2020. Recruitment of participants included healthy elderly, CAD, and HTN patients who volunteered for health examinations in Taipei City and the surrounding rural area in Taiwan. The exclusion criteria were rheumatic valvular disease, severe mitral or aortic regurgitation, significant aortic stenosis (peak valvular systolic gradient of ≥40 mmHg), a fasting plasma glucose level of >126 mg/dL (7.0 mmol/L) or casual plasma glucose of >200 mg/dL (11.1 mmol/L), HbA1c level of >7%, thyroid disease, cerebrovascular accidents, malignancy, body mass index (BMI) of >30 kg/m^2^, subjects with a poor acoustic window or with echocardiographic evidence of heavy mitral annulus calcification, hemoglobin of ≤13 g/dL, albumin of ≤3.2 g/dL, or serum creatinine of ≥1.4 mg/dL. All subjects underwent 2D echocardiography and tissue Doppler imaging (TDI) examinations. This study was approved by the Ethics Committee and Institutional Review Board (IRB) on Human Research of the Medical Research Department of the National Taiwan University Hospital, Taipei, Taiwan. All subjects provided written informed consent before participating in the study.

**Figure 1:**
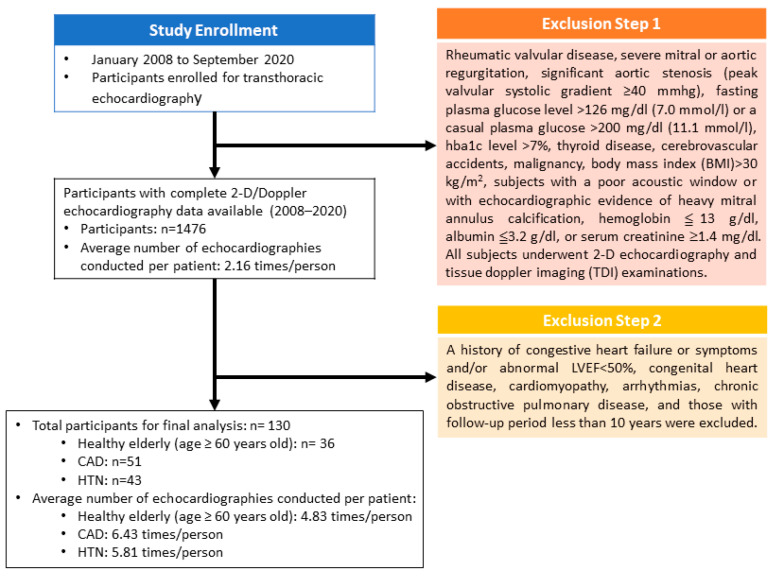
Flowchart of study design, data source, participants’ eligibility and exclusion, and available data numbers.

In this study, we planned to investigate the sequential changes in LV diastolic function with a preserved left ventricular ejection fraction (LVEF) in healthy elderly individuals (>60 years) and patients with CAD and HTN. Therefore, a history of congestive heart failure or symptoms and/or abnormal LVEF of <50%, congenital heart disease, cardiomyopathy, arrhythmias, chronic obstructive pulmonary disease, and those with a follow-up period of less than 10 years were excluded. A total of 130 participants (36 healthy elderly (aged ≥ 60 years old), 51 with CAD, and 43 with HTN) and 752 echocardiography examinations were included in the final analysis (Figure 1).

### 2.2. Study Algorithms

An annual re-evaluation was arranged for each participant after the initial enrollment. Blood pressure (BP) measurements, medications, laboratory, and echocardiography examinations were checked annually during the follow-up period. In order to retain the maximal recruitment of this long-duration study, several strategies were performed to lower the attrition rates. First, the environment for the echocardiographic examination was user-friendly. In particular, for the elderly participants, there was a full-time assistant to accompany them during the course of the echocardiographic measurements. Second, the appointment times were flexible during the course of follow-up. Third, participants were oriented to their roles, the study demands, and research responsibility. Finally, the results of the examinations were clearly explained to the participants as incentives and feedback for their participation.

### 2.3. Grouping

The key goal of this study was to evaluate the evolutional change in LV diastolic function between the subjects with CAD and HTN. Therefore, the aging-related decrease in LV diastolic function should be considered. First, healthy elderly participants without any systemic diseases were enrolled. They were categorized as the control group in comparison with the CAD and HTN groups.

The CAD subjects were screened according to their clinical symptoms, physical examinations, resting electrocardiography, exercise stress electrocardiography, myocardial perfusion imaging, or electron-beam computed tomography (CT). They all accepted coronary angiography for the final diagnosis of CAD, and the results were evaluated by two cardiologists. In addition to lifestyle modifications (e.g., weight control, physical activity, and avoidance of exposure to tobacco smoke), the CAD patients were all under regular pharmacotherapy for ischemic heart diseases [5,6,7].

For the HTN subjects, the definition of HTN was BP of >140/90 mmHg or those taking HTN medication. The on-treatment target BP was less than 130/80 mm Hg [8,9,10]. The long-term anti-HTN treatment and reasonable BP control depended on the patient’s clinical condition and were adjusted according to the Joint National Conference VII guidelines [8,9,10]. The target BP was also updated by the clinicians during this period [8,11]. The HTN patients also were prescribed significant lifestyle modifications (e.g., the DASH (Dietary Approaches to Stop Hypertension) Diet and weight control (weight aim for BMI of 18.5–24.9 kg/m^2^) and physical activity) during this period [11].

### 2.4. Two-Dimensional Echocardiography and Tissue Doppler Imaging

Comprehensive echocardiographic measurements using 2D echocardiography and TDI were performed with Vivid S5 (GE Company, Vingmed, Horten, Norway) equipped with a 1.7/3.4 MHz imaging transducer, with standardized imaging acquisition from key cardiac views, as recommended for appropriate cardiac chamber size and dimensions for M-mode and 2D or Doppler quantification according to the American Society of Echocardiography guidelines [12,13,14]. The echocardiographic method for measurement of LVEF was the apical biplane method of discs (the modified Simpson’s rule) [12,13,14]. 

The transmitral Doppler flow velocity was measured using a 5 mm sample volume placed at the tips of the mitral leaflets in passive end expiration. A standardized loop of 10 cardiac cycles was downloaded to a computer for offline analysis of the early filling (E-wave) and late filling (A-wave) phases [12,13,14]. Tissue Doppler velocities were then acquired at the level of the septal and lateral mitral annulus. The early diastolic e′ velocity and a′ velocity at the septal and lateral annular site were measured. The e′/a′ and the E/e′ ratios were calculated according to the guidelines from the American Society of Echocardiography [12,13,14]. To obtain accurate data while performing Doppler measurements, the septal and lateral walls were highlighted in the apical 4-chamber view. Using a pulse-wave Doppler, a sample volume of 4.0 mm was placed at the septal side of the mitral annulus. This process was then repeated for the lateral side of the mitral annulus. The average septal or lateral e′ was calculated [14]. Baseline clinical information, biochemical data, and medical histories were also collected.

### 2.5. Score Calculation by the Novel Integrated Score Index

The diastolic score was calculated to evaluate the association of LV diastolic function with cardiac aging, CAD, and HTN. This score index was based on the results of the echocardiography with a score of 1 for an E/A ratio of <1, a score of 1 for a septal e′/a′ ratio of ≤0.8, a score of 2 for a lateral e′/a′ ratio of ≤1, a score of 2 for a septal E/e′ ratio of ≥10–15, a score of 3 for a lateral E/e′ ratio of ≥8–15, and a score of 1 for a deceleration time of >240 ms. The sum of each score was considered as the final value for the score index. Either a septal or a lateral E/e′ ratio of >15 was given a total score of 10, regardless of the other measurements [15].

### 2.6. Statistical Analyses

Continuous data are expressed as the mean ± standard deviation (SD). Differences between the continuous data of groups were determined by performing one-way analysis of variance (ANOVA) followed by the Fisher’s Least Significant Difference (LSD) post hoc test for multiple paired comparisons. Even though the next scheduled appointment was arranged after each examination, some participants missed the annual evaluations unexpectedly. Hence, we chose linear mixed models (LMM) to analyze the longitudinal data of each group [16]. There were nested and repeated measurements within individual groups. The longitudinal change in diastolic function with aging among groups was compared by generalized estimating equation (GEE) analysis [17]. A two-tailed *p*-value of <0.05 was considered statistically significant. Statistical analysis was performed by SPSS software version 24.0 (IBM Corp., Armonk, NY, USA).

## 3. Results

### Basic Characteristics and Demographics of Echocardiography

This study was conducted from January 2008 to September 2020. The total follow-up time was 5751 person-years; the mean follow-up time was 7.68 person-years. The time of follow-up examination was arranged annually after the first echocardiographic examination. From 2008 to 2020, 1476 patients were enrolled in the study, and 3181 follow-up echocardiographies were collected. However, some participants were lost to regular follow-up, and data with less than a 10-year follow-up period were excluded. At the end of the study, a total of 130 participants (36 healthy elderly (age ≥ 60 years old), 51 with CAD, and 43 with HTN) and 752 echocardiography examinations remained for final analysis (Supplementary Table S1).

Generally, the thickness of the left ventricular wall, such as the interventricular septum (IVS) and left ventricular posterior wall (LVPW), was almost the same between the groups at enrollment (Table 1). These diastolic parameters including the E/A ratio, deceleration time (DT), septal e′/a′ ratio (Sep e′/a′), lateral e′/a′ ratio (Lat e′/a′), septal E/e′ ratio (Sep E/e′), and lateral E/e′ ratio (Lat E/e′) were not significantly different between the groups at baseline (Table 1). The E/e′ ratio, either septal or lateral, was less than 15 among all groups at the beginning (Table 1).

Cardiac function was further evaluated by the integrated score index and the New York Heart Association (NYHA) class [15,18]. Either the diastolic or clinical function was similar between the groups at baseline (Table 1). Renal function was evaluated by the serum creatinine (Cr) level and estimated glomerular filtration (eGFR). Renal function was initially within normal limits (Table 1).

Basic medications, including beta-blockers (β-blocker), calcium-channel blockers (CCB), angiotensin-converting enzyme inhibitors/angiotensin receptor blockers (ACEI/ARB), and Spironolactone were categorized and analyzed. Compared with the elderly and CAD subjects, the HTN subjects had a higher usage of CCB and ACEI/ARB medication upon enrollment (*p* < 0.001) (Table 1).

The longitudinal change in cardiac function with aging during this period for each group (elderly, CAD, and HTN) was individually analyzed by the linear mixed model (LMM) method. The results for each group are summarized in Table 2. In brief, the wall thickness of the LV (e.g., IVS or PW) among the HTN subjects did not change with aging, whereas it did increase with aging for the elderly and CAD subjects (*p* < 0.001) (Table 2). The LVEF was used to evaluate the LV systolic function; it decreased with aging among all three groups (all *p* < 0.05) (Table 2). Diastolic function was measured by the diastolic parameters (e.g., E/A, DT, Sep e′/a′, Lat e′/a′, Sep E/e′, and Lat E/e′) and decreased with aging in most groups (*p* < 0.001) (Table 2). Interestingly, only the values of the Lat e′/a′ (*p* = 0.07) and Lat E/e′ (*p* = 0.28) did not change with aging for the HTN subjects (Table 2). For the elderly and CAD subjects, the integrated score index increased 0.2 per year and 0.15 per year, respectively (all *p* < 0.001) (Table 2). However, the integrated score index only increased 0.06 per year (*p* = 0.03) in the HTN subjects (Table 2). Compared with the elderly and CAD groups, it seems that the diastolic function was preserved in the HTN subjects during this period.

In order to compare the longitudinal change in diastolic function with aging and clarify the possible factors associated with the change in diastolic function between each group, the GEE analysis was performed for two-group comparisons. The results are summarized in Table 3. In general, there was no significant difference between the elderly and CAD groups in systolic and diastolic function during the study period (Table 3). Even the integrated score index and the functional NYHA class were almost the same (Table 3). For the HTN group, the LV thickness (IVS and PW, all *p* < 0.05) and LV diastolic parameters (Sep E/e′ and Lat E/e′, all *p* < 0.05) were different both within the elderly and HTN comparison and the CAD and HTN comparison (Table 3). This phenomenon was also noted for the integrated score index (all *p* = 0.01) and the functional NYHA class (all *p* < 0.05) (Table 3). Therefore, diastolic function proved to be preserved in the HTN subjects during follow-up in comparison with elderly and CAD subjects after the two-group comparisons.

The lab data and mediation were also analyzed by the GEE method to investigate the possible factors contributing to these results. However, there were no obvious differences in lab data (hemoglobulin and renal function) or type of medication used in these groups (e.g., β-blocker, CCB, ACEI/ARB, and Spironolactone) after the two-group comparisons (Table 3).

## 4. Discussion

This was a very long-term real-world observational study; therefore, participants lost-to-follow-up and selection bias were inevitable during such a long period of time (more than one decade). Additionally, a relevant proportion of patients in the CAD group may also have suffered HTN after enrollment. Therefore, we hypothesized that the observed difference would tend toward the null in this intention-to-treat study. If there was any evolutional change in diastolic function between these groups at the end of the study, the real difference between them would be larger than our expectation.

### 4.1. The Main Findings of This Study 

There were three main findings from our study, providing new evidence for possible clinical application. First, diastolic function was preserved in the HTN subjects with aggressive treatment in comparison with the elderly and CAD participants. Second, specific parameters such as the Lat E/e′ and the integrated score index seemed to be able to detect the difference in aging effects on the LV diastolic function among these groups. Finally, aggressive BP control appeared to be an effective method of reducing the risk of HFpEF during this longitudinal study.

### 4.2. The Clinical Parameters for Detecting LV Diastolic Function with Aging

In a previous study, the integrated score index was shown to discriminate healthy subjects from patients with LV diastolic dysfunction (e.g., CAD, HTN, and hypertrophy cardiomyopathy) [15]. Compared with those with impaired diastolic function, the healthy subjects obtained lower integrated scores (score of <4) [15]. In this study, we used the common echocardiographic parameters and the integrated score index to evaluate the change in LV diastolic function with the aging process.

Based on the echocardiographic pattern, most of the common diastolic parameters (e.g., E/A, DT, Sep e′/a′, Lat e′/a′, Sep E/e′, and Lat E/e′) decreased with aging in each group (*p* < 0.001) (Table 2). Only the values of Lat e′/a′ (*p* = 0.05) and Lat E/e′ (*p* = 0.23) detected that LV diastolic function was preserved in the HTN subjects during this period (Table 2). The same results were noted after analyzing the integrated score index. The integrated score index increased 0.2 score per year and 0.15 score per year for the elderly and CAD groups, respectively (all *p* < 0.001) (Table 2). In contrast, the integrated score index increased just 0.06 score per year in the HTN participants (*p* = 0.02) (Table 2). These echocardiographic findings were also compatible with the results of the functional class evaluated by the NYHA class (all *p* < 0.05) (Table 3).

In previous studies, the correlation of the E/e′ ratio with pulmonary capillary wedge pressure was well established in patients with LV systolic dysfunction [19]. However, the evidence to support using the E/e′ ratio to estimate LV diastolic dysfunction in patients with preserved LVEF was still insufficient [19]. From our results, the integrated score index could reveal the change in diastolic function with preserved LVEF with a clear and accurate number. Therefore, we suggest combining both the E/e′ ratio and the integrated score index together to evaluate the change in LV diastolic function in subjects with preserved LVEF in real-world practice. These parameters may be useful for identifying patients at increased risk of developing incidental HF at a preclinical level.

### 4.3. The Change in LV Diastolic Function with Aging

In this study, we compared the change in LV diastolic function with aging among three groups (elderly, CAD, and HTN). As discussed above, both CAD and HTN subjects were prescribed lifestyle modifications and pharmacological therapy while enrolled in this study. However, the diastolic function was preserved in the HTN group during this period. It seems that the aging effects on diastolic function in the subjects with preserved LVEF were different between diseases.

Previously, Kuznetsova et al. reported a longitudinal follow-up of left ventricular diastolic function in the general population [4]. From their study, 828 cases were enrolled, and 650 cases with an average age of 50.7 years old were available for final analysis after 4.7 years of follow-up [4]. They found the baseline age and the change in SBP over time were associated with the worsening of diastolic function [4]. Different from their study, CAD patients were specifically enrolled in our study for comparison. Our follow-up time was more than 10 years, more than twice the length of their study [4]. The average age in our study was more than one decade older than theirs [4]. Furthermore, the echocardiographic examinations were arranged annually for our participants, rather than at the 5-year interval of their study. With more frequent echocardiography examinations for our patients, we could present more details on the evolutional changes in the diastolic function with the aging process [4]. Both of these studies encountered the same problems of participants who were lost-to-follow-up [4]. This problem is inevitable and hard to address in long-term longitudinal observational studies. Therefore, our study is by far the longest follow-up study, with the oldest participants enrolled, that compares the association of diastolic function between HTN and CAD patients during the aging process [4].

### 4.4. The Aging Effects on the Diastolic Function between the Elderly and HTN Groups

Compared with the elderly group, the diastolic function of the HTN group was preserved after 12 years. We noticed that the evolutional change in SBP with aging in the HTN and elderly groups was different (*p* = 0.18 and <0.001, respectively) (Table 2). This might be due either to the aging-associated physiological changes between these two groups or to the clinical effects after ongoing HTN treatments. As Kuznetsova et al. suggested, the change in SBP was associated with worsening diastolic function [4]. This could prove that the HTN patients had good BP control and that aggressive HTN treatments might slow the deterioration of diastolic function during the aging process. Interestingly, the baseline DBP was significantly different among the three groups in our study (elderly, CAD, and HTN = 72.14 ± 8.01, 77.22 ± 9.02, and 81.35 ± 9.27, respectively, *p* < 0.001) (Table 1). From the suggestions of Kuznetsova et al., the baseline DBP was associated with worsening diastolic function [4]. Therefore, it seems that the benefits of long-term HTN treatments might alleviate the pernicious consequences related to baseline DBP. 

The previous Aspirin in Reducing Events in the Elderly (ASPREE) trial also provided similar insights to the present study [20]. In the ASPREE study, participants were generally healthy individuals aged 65 years or older. Among them, 74% had HTN and well-controlled blood pressure [21]. The prior ASPREE analyses revealed that high visit-to-visit blood pressure variability was associated with increased risk of cardiovascular events, and, therefore, deteriorated cardiac function as a consequence [22]. The present study implies the importance of blood pressure control during the aging process, which was consistent with the ASPREE analyses [22]. 

### 4.5. The Aging Effects on the Diastolic Function between the CAD and HTN Groups

In this study, the participants in the CAD and HTN groups were prescribed lifestyle modifications and regular pharmacotherapy. The evolutional change in SBP with aging was significant in the CAD group (*p* < 0.001) (Table 2) rather than the HTN group (*p* = 0.18) (Table 2). This might be attributed to the effects of HTN treatments. In this study, the types of medications were also analyzed (e.g., β-blocker, CCB, ACEI/ARB, and Spironolactone), and there was no significant difference among the medications between the CAD and HTN groups (Table 3). In a previous study, multiple anti-HTN drugs were shown to be beneficial for cardiac function in large randomized trials [23]. Diuretics, ACEI/ARB, and CCB appear to be the most effective agents at reducing LV hypertrophy and HF risk as compared with other agents, such as alpha-blockers [23]. Therefore, aggressive BP control might be superior to medication type, but more studies are needed to address this issue.

## 5. Limitations

This was a one-center observational study with more than one decade of follow-up. There were some inevitable limitations. In this study, initially, the treatments for HTN were according to JNC 7. The target value of BP control was adjusted and updated by the clinicians according to the patient’s clinical condition during this period, which might have influenced the results. The selection bias and the small study patient group were the main negative parameters that could have affected our results. The limited sample size for this type of longitudinal analysis and the large number of covariables compared should be taken into consideration. Specific parameters for special populations such as HTN and CAD patients could provide more information about this issue. Finally, this study began more than 10 years ago, so the recommendations for the evaluation of left ventricular diastolic function by echocardiography were from 2009.

## 6. Conclusions

In this more than 12-year study, LV diastolic function decreased gradually with increasing age among all groups. There was no significant difference between the elderly and CAD groups in both systolic and diastolic function during this period. In contrast, the LV diastolic function was preserved in the HTN group in comparison with the elderly and CAD participants. We suggest that aggressive BP control could slow the LV diastolic function deterioration that commonly occurs with the aging process.

## Figures and Tables

**Table 1:** Baseline characteristics of each group.

	Elderly	CAD	HTN	
Cases (M/W)	*n* = 36 (12/24)	*n* =51 (42/9)	*n* = 43 (23/20)	
Diastolic Dysfunction Grade (normal/1/2/3) [12]	28/3/5/0	7/6/38/0	4/10/29/0	
	Mean ± SD	Mean ± SD	Mean ± SD	*p*-Value
Age (yrs)	79.39 ± 9.51	68.31 ± 12.09	67.51 ± 5.92	<0.001
BMI (kg/m^2^)	23.32 ± 2.67	25.17 ± 2.98	24.84 ± 2.98	0.01
SBP (mmHg)	132.75 ± 15.80	134.20 ± 14.70	138.93 ± 17.33	0.19
DBP (mmHg)	72.14 ± 8.01	77.22 ± 9.02	81.35 ± 9.27	<0.001
HR (beats/min)	69.31 ± 9.56	68.55 ± 8.18	66.40 ± 8.26	0.29
EDD (mm)	4.43 ± 0.53	4.77 ± 0.59	4.77 ± 0.43	0.01
LA (mm)	3.39 ± 0.50	3.75 ± 0.55	3.47 ± 0.70	0.01
IVS (mm)	0.97 ± 0.23	1.03 ± 0.27	0.98 ± 0.16	0.39
PW (mm)	0.90 ± 0.18	0.94 ± 0.17	0.90 ± 0.15	0.48
LVEF (%)	61.75 ± 6.25	60.46 ± 8.23	62.54 ± 7.86	0.41
E/A	0.83 ± 0.25	0.96 ± 0.35	0.92 ± 0.25	0.11
DT (ms)	233.21 ± 45.42	233.06 ± 52.60	232.69 ± 49.93	1
Sep e′/a′	0.65 ± 0.20	0.67 ± 0.22	0.65 ± 0.15	0.84
Lat e′/a′	0.77 ± 0.31	0.80 ± 0.28	0.78 ± 0.24	0.89
Sep E/e′	12.73 ± 4.22	12.73 ± 3.91	12.37 ± 3.93	0.89
Lat E/e′	9.54 ± 2.82	9.32 ± 3.40	9.21 ± 3.00	0.9
Score	7.31 ± 2.97	6.82 ± 3.17	7.19 ± 2.88	0.73
NYHA	1.11 ± 0.32	1.08 ± 0.27	1.00 ± 0.00	0.1
Hb (g/dL)	12.72 ± 1.54	14.35 ± 1.64	13.38 ± 1.30	0.01
Cr (mg/dL)	0.84 ± 0.22	0.98 ± 0.20	0.88 ± 0.21	0.03
K (mmol/L)	4.15 ± 0.50	4.05 ± 0.39	4.32 ± 0.29	0.1
eGFR (mL/min/1.73 m^2^)	76.24 ± 21.17	79.89 ± 19.04	82.15 ± 15.19	0.64
β-blocker	0.19 ± 0.40	0.35 ± 0.48	0.35 ± 0.48	0.23
CCB	0.53 ± 0.51	0.39 ± 0.49	0.77 ± 0.43	<0.001
ACEI/ARB	0.42 ± 0.50	0.57 ± 0.50	0.86 ± 0.35	<0.001
Spironolactone	0.00 ± 0.00	0.08 ± 0.27	0.12 ± 0.32	0.12

CAD, coronary artery disease; HTN, hypertension; M, male; F, female; BMI, body mass index; SBP, systolic blood pressure; DBP, diastolic blood pressure; HR, heart rate; EDD, end diastolic dimension; LA, left atrium; IVS, interventricular septum; PW, posterior wall; LVEF, left ventricular ejection fraction; E/A ratio, the ratio of mitral inflow E-wave/A-wave velocity; DT, E-wave deceleration time; Sep e′/a′, the ratio of septal mitral annulus early diastolic velocity/mitral annulus late diastolic velocity; Lat e′/a′, the ratio of lateral mitral annulus early diastolic velocity/mitral annulus late diastolic velocity; Sep E/e′, the ratio of septal early diastolic flow velocity/mitral annulus early diastolic velocity; Lat E/e′, the ratio of lateral early diastolic flow velocity/mitral annulus early diastolic velocity; NYHA, New York Heart Association; Hb, hemoglobulin; Cr, creatinine; K, potassium; eGFR, estimated glomerular filtration; β-blocker, beta-blocker; CCB, calcium-channel blocker; ACEI/ARB, angiotensin-converting enzyme inhibitors/angiotensin receptor blocker.

**Table 2:** Longitudinal change with age in the elderly, CAD, and HTN groups, using a linear mixed model (LMM) analysis.

	Elderly		CAD		HTN	
	Change with Age (per Year)	*p*-value	Change with Age (per Year)	*p*-Value	Change with Age (per Year)	*p*-Value
BMI (kg/m^2^)	0.038	<0.001	−0.01	0.17	−0.083	0.01
SBP (mmHg)	0.5	<0.001	0.212	<0.001	0.212	0.18
DBP (mmHg)	−0.308	<0.001	−0.363	<0.001	−0.382	<0.001
HR (beats/min)	0.153	<0.001	0.002	0.97	0.345	<0.001
EDD (mm)	−0.006	<0.001	−0.007	0.01	0.005	0.24
LA (mm)	0.009	0.01	−0.002	0.46	0.014	0.01
IVS (mm)	0.008	<0.001	0.002	0.11	0.001	0.65
PW (mm)	0.004	<0.001	0.002	<0.001	0	0.85
LVEF (%)	−0.191	0.01	−0.111	<0.001	−0.13	<0.001
E/A	−0.003	<0.001	−0.012	<0.001	−0.011	<0.001
DT (ms)	0.501	<0.001	2.217	<0.001	1.661	<0.001
Septal e′/a′	−0.004	<0.001	−0.006	<0.001	−0.006	<0.001
Lateral e′/a′	−0.005	<0.001	−0.011	<0.001	0.004	0.05
Septal E/e′	0.248	<0.001	0.183	<0.001	0.084	0.01
Lateral E/e′	0.147	<0.001	0.167	<0.001	0.032	0.23
Score	0.2	<0.001	0.152	<0.001	0.057	0.02

CAD, coronary artery disease; HTN, hypertension; BMI, body mass index; SBP, systolic blood pressure; DBP, diastolic blood pressure; HR, heart rate; EDD, end diastolic dimension; LA, left atrium; IVS, interventricular septum; PW, posterior wall; LVEF, left ventricular ejection fraction; E/A ratio, the ratio of mitral inflow E-wave/A-wave velocity; DT, E-wave deceleration time; Sep e′/a′, the ratio of septal mitral annulus early diastolic velocity/mitral annulus late diastolic velocity; Lat e′/a′, the ratio of lateral mitral annulus early diastolic velocity/mitral annulus late diastolic velocity; Sep E/e′, the ratio of septal early diastolic flow velocity/mitral annulus early diastolic velocity; Lat E/e′, the ratio of lateral early diastolic flow velocity/mitral annulus early diastolic velocity.

**Table 3:** Comparison of the 12-year trend among groups, using the generalized estimating equation (GEE) analysis.

	Elderly vs. CAD		Elderly vs. HTN		CAD vs. HTN	
	The β-Value Is Based on the Ratio of Elderly/CAD		The β-Value Is Based on the Ratio of Elderly/HTN		The β-Value Is Based on the Ratio of CAD/HTN	
	Interaction term (β-Value)	*p*-Value	Interaction term (β-Value)	*p*-Value	Interaction Term (β-Value)	*p*-Value
BMI (kg/m^2^)	0.005	0.88	0.041	0.37	0.036	0.37
SBP (mmHg)	0.411	<0.001	0.462	0.02	0.050	0.76
DBP (mmHg)	0.075	0.59	0.078	0.62	0.003	0.97
HR (beats/min)	0.036	0.70	−0.242	0.04	−0.278	<0.001
EDD (mm)	0.003	0.58	−0.005	0.41	−0.008	0.13
LA (mm)	0.008	0.11	−0.002	0.79	−0.011	0.15
IVS (mm)	0.005	0.03	0.008	<0.001	0.003	0.08
PW (mm)	0.004	0.01	0.006	<0.001	0.003	0.03
LVEF (%)	−0.002	0.98	0.174	0.01	0.176	0.01
E/A	0.001	0.74	0.003	0.49	0.002	0.62
DT (ms)	−0.721	0.08	−0.708	0.20	0.013	0.98
Sep e′/a′	−0.003	0.25	−0.002	0.48	0.001	0.65
Lat e′/a′	−0.003	0.51	−0.002	0.73	0.001	0.69
Sep E/e′	0.044	0.23	0.152	<0.001	0.109	0.01
Lat E/e′	0.006	0.87	0.147	<0.001	0.142	<0.001
Score	0.027	0.42	0.111	0.01	0.085	0.01
NYHA	−0.004	0.40	0.010	0.03	0.014	<0.001
Hb (g/dL)	−0.020	0.37	−0.021	0.53	−0.001	0.98
Cr (mg/dL)	−0.006	0.16	−0.003	0.55	0.003	0.35
K (mmol/L)	−0.033	<0.001	−0.014	0.08	0.018	0.01
eGFR (mL/min/1.73 m^2^)	0.932	0.03	0.571	0.22	−0.361	0.17
β-blocker	−0.019	0.45	−0.007	0.83	0.012	0.63
CCB	−0.088	<0.001	−0.076	0.04	0.012	0.69
ACEI/ARB	−0.074	0.02	−0.065	0.12	0.009	0.76
Spironolactone	0.025	0.61	0.026	0.63	0.001	0.98

CAD, coronary artery disease; HTN, hypertension; M, male; F, female; BMI, body mass index; SBP, systolic blood pressure; DBP, diastolic blood pressure; HR, heart rate; EDD, end diastolic dimension; LA, left atrium; IVS, interventricular septum; PW, posterior wall; LVEF, left ventricular ejection fraction; E/A ratio, the ratio of mitral inflow E-wave/A-wave velocity; DT, E-wave deceleration time; Sep e′/a′, the ratio of septal mitral annulus early diastolic velocity/mitral annulus late diastolic velocity; Lat e′/a′, the ratio of lateral mitral annulus early diastolic velocity/mitral annulus late diastolic velocity; Sep E/e′, the ratio of septal early diastolic flow velocity/mitral annulus early diastolic velocity; Lat E/e′, the ratio of lateral early diastolic flow velocity/mitral annulus early diastolic velocity; NYHA, New York Heart Association; Hb, hemoglobulin; Cr, creatinine; K, potassium; eGFR, estimated glomerular filtration; β-blocker, beta-blocker; CCB, calcium-channel blocker; ACEI/ARB, angiotensin-converting enzyme inhibitors/angiotensin receptor blocker.

## Data Availability

All data relevant to the study are included in the article.

## Supplementary Materials

The following are available online at https://www.mdpi.com/article/10.3390/jpm12030415/s1, Supplementary Table S1. Longitudinal echocardiography examination times during 12-year period.
