# Supplementary material for: The Sequential Change in Left Ventricular Function among Various Cardiovascular Diseases: A 12-Year Study
Source: J Pers Med. 2022 Mar 7;12(3):415. doi: 10.3390/jpm12030415 (PMC8950536; doi:10.3390/jpm12030415)
Supplement: Supplementary file 1 [file jpm-12-00415-s001.zip › jpm-1567244-supplementary.pdf]

Supplementary Table S1. Longitudinal echocardiography examination times during 12-year period.

	Times (M)	Times (F)	Total Times
Elderly	63	111	174
CAD	278	50	328
HTN	137	113	250
Total Times	478	274	752

M, male; F, female; CAD, coronary artery disease; HTN, hypertension.
